# Cancer Vaccines: A Promising Therapeutic Strategy in Advanced Solid Tumors

**DOI:** 10.3390/vaccines13060591

**Published:** 2025-05-30

**Authors:** Simona Caridi, Valeria Maccauro, Lucia Cerrito, Gianluca Ianiro, Maria Pallozzi, Leonardo Stella, Antonio Gasbarrini, Francesca Romana Ponziani

**Affiliations:** 1 Liver Unit, Centro Malattie dell’Apparato Digerente (CEMAD), Medicina Interna e Gastroenterologia, Fondazione Policlinico Universitario Gemelli IRCCS, 00168 Rome, Italy; simona.caridi.97@gmail.com (S.C.); valeriamaccauro@gmail.com (V.M.); lucia.cerrito@policlinicogemelli.it (L.C.); mariapallozziucsc@gmail.com (M.P.); leonardo.stella@guest.policlinicogemelli.it (L.S.); antonio.gasbarrini@unicatt.it (A.G.); 2 Department of Medical and Surgical Sciences, UOC Gastroenterologia, Fondazione Policlinico Universitario A. Gemelli IRCCS, 00168 Rome, Italy; gianluca.ianiro@policlinicogemelli.it; 3 Dipartimento di Medicina e Chirurgia Traslazionale, Università Cattolica del Sacro Cuore, 00168 Rome, Italy

**Keywords:** immunotherapy, therapeutic vaccines, tumor microenvironment (TME), tumor antigens, advanced solid tumors, immune checkpoint inhibitors (ICIs)

## Abstract

Recent advancements in understanding how cancer cells evade immune recognition have led to significant progress in cancer immunotherapy. Therapeutic cancer vaccines hold great promise due to their safety, specificity, and ability to establish lasting immune memory, serving as an effective immunotherapy either alone or in combination with other treatments in clinical research. Cancer vaccines aim to restore the host’s innate and adaptive anti-cancer immune responses by stimulating antigen-presenting processes and reversing the immunosuppressive environment that facilitates tumor immune evasion and metastasis. Although in clinical studies cancer vaccines have been observed to not effectively induce tumor regression, they can enhance local immune responses in combination with other immunotherapeutic agents, such as immune checkpoint inhibitors (ICIs), thus delaying cancer recurrence and prolonging overall survival in advanced tumor settings.

## 1. Introduction

Nowadays, solid tumors are diagnosed in advanced metastatic stages, as surgery and chemotherapy may not be suitable therapeutic options to prolong patient overall survival. In this regard, immunotherapy, an innovative technique specifically targeting cancer cells by fostering the patient’s own defenses, has been developed with promising results.

Cancer immunotherapy enhances the activity of the patient’s immune cells against tumors using innovative approaches, including adoptive cell therapy (ACT), immune checkpoint inhibition (ICIs), and cancer vaccines [1].

Tumors often downregulate immune responses through the tumor microenvironment (TME), which involves immunosuppressive cells such as regulatory T cells (Tregs) and myeloid-derived suppressor cells (MDSCs). Re-activating the immune system typically requires exogenous antigen-presenting cells (APCs), which present tumor-associated antigens to cytotoxic (CD8+) and helper (CD4+) T cells via major histocompatibility complex (MHC) molecules [2].

**Figure 1:**
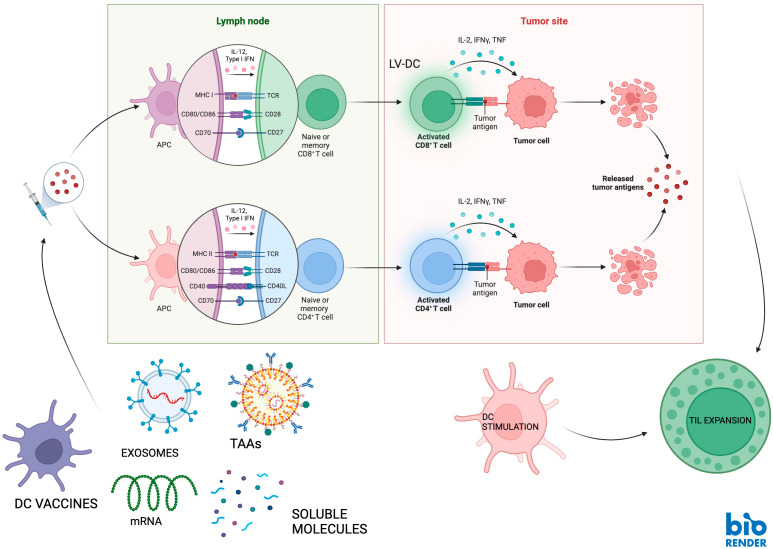
Cancer vaccines and their mechanisms of action. Cancer vaccines can stimulate local host adaptive anti-tumor immune responses. Vaccines carrying tumor-associated antigens are recognized by DCs at the tumor site. Then, activated DCs migrate through the lymphatic vessels to the T-cell zones of lymphoid tissues. There, they stimulate T lymphocytes by simultaneously expressing costimulatory molecules, thus creating CTLs specific for tumor-associated antigens presented on the surfaces of cancer cells via MHC class I molecules to destroy tumor cells. Meanwhile, naïve CD4+ T cells differentiate into effector cells, initiating a B cell-mediated, TAA/TSA-specific antibody response. Abbreviations: antigen-presenting cell (APC), major histocompatibility complex (MHC), dendritic cell (DC), tumor-infiltrating lymphocyte (TIL), T cell receptor (TCR), tumor-associated antigen (TAA), messenger RNA (mRNA).

Once tumor-associated antigens are recognized by dendritic cells (DCs) at the tumor site, these cells migrate through the lymphatic vessels to the T-cell zones of lymphoid tissues. There, they stimulate T lymphocytes by simultaneously expressing costimulatory molecules. DC-primed CD8+ cytotoxic T lymphocytes (CTLs) can identify tumor-associated antigens presented on the surface of cancer cells via MHC class I molecules and subsequently destroy these cells. Meanwhile, naïve CD4+ T cells differentiate into effector cells, initiating a B cell-mediated, TAA-specific antibody response [3] (Figure 1).

Solid tumors, however, have developed various mechanisms to evade the immune response. Factors contributing to the immunosuppressive TME include hypoxia, an altered extracellular matrix, neoangiogenesis, cancer-associated fibroblasts, Tregs, MDSCs, and tumor-associated M2 macrophages (TAMs). These elements collectively impair the effectiveness of immunotherapy [4]. To counteract this mechanism, new strategies are being developed to restore the anti-cancer immune response and enhance the efficacy of conventional immunotherapies.

Cancer vaccines utilize cancer-specific antigens to stimulate the immune system to counteract tumors by inducing a robust and long-lasting antigen-specific expansion of both CD4+ and CD8+ T cells [5]. These vaccines not only reinvigorate pre-existing immune responses, but also initiate new immune reactions against tumors [6].

In this review we aim to analyze the most important cancer vaccine types currently or potentially used as immunotherapy agents alone or in combination with other immunomodulants for advanced solid tumors.

## 2. Cancer Vaccines as “Antigen-Specific Target Therapy”

The effectiveness of cancer vaccines is based on the stimulation of an antigen-specific immune response targeted to induce tumor regression and to produce a sustainable memory immune response [7]. These vaccines are designed to activate the host’s T cells against specific antigens on cancer cells surfaces, which can be divided into tumor-associated antigens (TAAs) and tumor-specific antigens (TSAs, also called “neoantigens”); while the first ones are aberrant molecules self-expressed on cancer cells’ surfaces, the second ones are, instead, exclusively produced by tumor cells from non-driver genomic mutations unique to tumor cells [8,9].

As an example of TAAs, melanocyte differentiation proteins (e.g., gp100, tyrosinase) [10] and Human epidermal growth factor receptor 2 (HER2) [11], as well as Cancer-testis antigens, including New York esophageal squamous cell carcinoma 1 (NY-ESO-1) and the melanoma antigen gene (MAGE) protein family [12], have been used as cancer vaccines in many clinical trials to elicit responses against multiple class I and class II MHC-restricted epitopes.

However, despite the fact that cancer vaccines could effectively foster the host adaptive immune response against specific tumor epitopes, a low magnitude of CD8+ T cells and humoral agents was observed in clinical trials, which were ineffective in targeting tumor spread. This phenomenon may be explained via the cancer immunoediting processes, including antigen loss and the selection of non-immunogenic epitope variants (immunoselection), stimulated by vaccines targeting single specific epitopes on tumor cells [13,14].

The most important limitation of cancer vaccines stems largely from the challenge of identifying TAAs and neoantigens [9,15,16]. Tumor antigens can originate from various sources, including autologous modified tumor cells (known as autologous whole-cell vaccines) [17], killed tumor cells, tumor cell lysates, tumor-derived exosomes, tumor peptides and proteins, and neoantigens derived from genetic mutations in tumor-specific molecules, which trigger a cytotoxic T lymphocyte response [18]. Cancers characterized by a high tumor mutation burden (TMB) and a greater frequency of tumor neoantigens tend to exhibit higher rates of tumor infiltrating lymphocytes (TILs). These so-called “hot tumors” are generally more responsive to cytolytic T cell activity and immune checkpoint inhibitors [19,20]. However, the heterogeneity of cancer cells often results in the simultaneous expression of various antigen types, necessitating a combination of neoantigens to elicit an effective immune response.

Tumor cells can employ several mechanisms to evade immune recognition while protecting healthy tissues from autoimmune damage. These include the inefficient processing and presentation of tumor antigens, the upregulation of negative costimulatory ligands that induce T cell anergy and expand the regulatory cell populations, the production of immunosuppressive molecules, such as Fas-L, TGF-β, CTLA-4, and PD-1/PD-L1, the increased expression of the immunosuppressive enzyme indoleamine 2,3-dioxygenase, the downregulation of MHC-I molecules on the cell surface, and the impairment of antigen presentation [21]. Combining neoantigen vaccines with ICIs has been proposed to enhance immunotherapy outcomes. Neoantigen-based vaccines can amplify neoAg-specific TIL populations, which, when reactivated through the checkpoint blockade, can effectively infiltrate the TME and destroy cancer cells.

In addition, a critical role of tumor-cognate CD4+ T cells has been hypothesized. In fact, they are supposed to either promote antigen-specific CD8+ T cell responses by enhancing dendritic cell activity as antigen-presenting cells, or mediate direct tumor killing independent of CD8+ T cells and stimulate other innate immune cells’ antitumor responses. Thus, the lack of tumor-cognate CD4+ T-cell responses may explain the limited anti-tumoral efficacy observed in vaccines restricted to epitopes stimulating CD8+T cells [22,23].

On the other hand, neoantigens represent a new field of interest for developing anti-cancer vaccines that overcome the limitation of central immune tolerance, especially when combined with immune checkpoint inhibitors in advanced solid different tumors (e.g., bladder cancer, melanoma, Non-Squamous-Cell Lung Cancer) [24,25]. In particular, this can involve the formulation of shared neoantigens, representing driver mutations expressed by all cells within a patient’s tumor, such as KRAS. Nevertheless, we still require future studies seeking to create vaccines based on multiepitope shared neoantigens [26].

## 3. Types of Cancer Vaccines

Cancer vaccines are categorized as either preventive or therapeutic. Preventive vaccines aim to protect individuals from certain cancers by inducing immune memory against specific cancer antigens. In contrast, therapeutic vaccines stimulate the immune system to enhance the activity of endogenous T cells against the immunogenic antigens expressed on tumor cell surfaces [5]. A distinct subgroup, known as immune-modulatory vaccines, works by activating anti-regulatory T cells (anti-Tregs) to counteract the immunosuppression mediated by regulatory immune cells [27].

Cancer vaccines can be developed through various approaches [28], and can be distinguished into the following categories (Table 1):

- Cell-based vaccines. These involve immune cells pre-loaded with cancer-specific antigens and subsequently administered to patients, either alone or in combination with other anti-cancer therapies;
- Protein/polypeptide vaccines. These utilize modified molecules capable of eliciting specific immune responses;
- Nucleic acid vaccines. These include DNA and RNA vaccines designed to express tumor antigens, as well as nanovaccines and tumor exosome-based vaccines.

### 3.1. Cell-Based Vaccines

Cell-based vaccines can be derived from autologous or allogeneic human cancer cell lines or tumor lysates, either loaded on dendritic cells or administered in adjuvants to promote dendritic cell activation in order to stimulate specific CD8+ and CD4+ T cell responses [7].

In the first subgroup, Melacine and Canvaxin have been used as two early allogeneic tumor cell-based vaccines for melanoma. For Melacine, a significant benefit in overall survival (OS) and an improvement in recurrence-free survival (RFS) within a subgroup expressing HLA-A2 and HLA-Cw3 were observed, thus imparting the hypothesis that genetic predisposition might influence the response to cancer vaccines [35].

For Canvaxin, composed of a mixture of irradiated whole-cell melanoma lines with bacillus Calmette–Guérin (BCG) as a vaccine adjuvant, no clinical benefit versus placebo has been observed in patients with advanced melanoma [36].

On the other hand, in the second subgroup of this category, DC vaccines have demonstrated the ability to stimulate antitumor defenses through a dual mechanism—by priming effector T cells against tumor antigens, and by enhancing natural killer (NK) cell activity via cytokine release and the expression of adhesion molecules such as CD155 [37]. The use of DC vaccines has shown potential in enhancing the efficacy of cytokine-induced killer cell immunotherapy for solid tumors, leading to prolonged survival and a strengthened immune response without inducing specific autoimmune side effects [38].

A notable example was represented by Sipuleucel-T, a DC vaccine containing prostate acid phosphatase (PAP) combined with granulocyte–macrophage colony-stimulating factor (GM-CSF), which has been approved by the U.S. Food and Drug Administration (FDA) for the treatment of metastatic castration-resistant prostate cancer [39]. In a neoadjuvant setting prior to radical prostatectomy, Sipuleucel-T significantly increased tumor-immune infiltration in surgical specimens compared to pre-treatment biopsies, and improved OS by four months compared to placebo [39,40]. Despite its effectiveness, this drug is no longer available; in fact, it has been retired due to its high price, complex way of administration, and uncertainty regarding its reimbursement. Furthermore, the vaccine’s supply was limited during the first year of launch due to limited manufacturing capacity [41,42]. Even though Sipuleucel-T is not currently used in clinical practice, the idea of targeting cancer tissue’s differentiated antigens despite the presence of nonmutated ones has been attracting scientific interest, as it could overcome the limit of cancer immunoediting and avoid off-target effects in future trials [39].

Further research on DC vaccines in triple-negative breast cancer has shown an enhanced immune response within the tumor microenvironment, characterized by increased tumor-infiltrating lymphocytes (TILs), elevated PD-L1 expression, and heightened peripheral immune activity, including enhanced lymphocyte proliferation, infiltration, IFN-γ production, and humoral responses [43].

Additionally, a phase I clinical trial involving patients with stage I-II cervical cancer evaluated subcutaneous injections of a DC vaccine carrying keyhole limpet hemocyanin and full-length HPV16/18 E7 antigens. The study demonstrated increased CD4+ T cell responses and a cervical cancer-specific humoral immune response [44].

Recent studies in metastatic melanoma have reported promising results derived from the combination of DC vaccines and ICIs, such as anti-PD-1 and anti-CTLA-4 antibodies, even in patients refractory to checkpoint blockade therapies [45,46,47]. Despite these advances, the impact of this combination on OS has not been consistently significant, and further evidence is required to validate the clinical application of this approach [48].

### 3.2. Protein/Polypeptide Vaccines

Peptide-based vaccines are specific, safe, and effective in producing a strong, adaptive anti-cancer immune response. The peptide-MHC and T cell receptor interactions are highly immunogenic and result in less ‘off-target’ toxicity and central tolerance. As an example, the Synthetic Long Peptide (SLP) vaccines are subunit vaccines made from peptides mimicking cancer antigens’ epitopes. They contain class-I and class-II MHC- restricted neoepitopes that help to stimulate both specific CD8+ T cell and CD4+ T cell responses. Immunogenicity increases with vaccines containing a huge amount of epitopes and recognition motifs. In fact, the use of long synthetic peptides of tumor antigens instead of short peptides has been observed to foster antigen presentation and tumor responses [14]. Short peptides (~9 aminoacid residues), instead, can be either easily digested by host’s enzymes, or loaded onto MHC-I molecules of non-professional APCs, leading to a wicker T cell response. Longer peptides (25–35 aminoacid residues), instead, are usually loaded onto professional APCs, in order to stimulate specific MHC-II T cell responses [29]. Two important clinical examples of this subgroup are a protein vaccine targeting the parathyroid hormone-related protein (PTHrP) in hypercalcemic solid tumors, and a combination of cancer peptides, called TAS0313, derived from eight disregulated cancer genes (EGFR, KUA, LCK, MRP3, PTHRP, SART2, SART3, and WHSC2) in glioblastoma [49]. Currently, a novel proteogenomic approach is under development to identify potential protein antigens (e.g., 19 tumor-specific peptides) for cancer vaccines in advanced colorectal cancer, but further evidence is needed before applying them in clinical practice [50].

### 3.3. Nucleic Acid-Based Vaccines

mRNA-based vaccines have gained significant attention due to their versatility and favorable safety profile. Unlike viral vector vaccines, mRNA vaccines are non-integrative, non-infectious, and readily modifiable. They are delivered via carrier molecules (often lipid nanoparticles) and do not elicit anti-vector immunity [30,51]. Upon administration at tumor sites, mRNA vaccines are recognized by Toll-like receptors (TLR7/8), which stimulate type I interferon (IFN) production, thereby enhancing DC-mediated antigen presentation to T cells and IL 12-expression [51]. Another advantage is that mRNA-encoded proteins can undergo post-translation modifications such as glycosylation, acetylation and methylation to produce tumor-specific folded proteins, which could in turn become antigens for cancer vaccines [14].

The clinical application of mRNA vaccines has shown remarkable promise, in particular in stage III and IV melanoma, as personalized RNA-based vaccines combined with PD-1 blockade therapy reduced metastasis rates and improved progression-free survival [52]. A phase Ib trial demonstrated durable neoantigen-specific CD4+ and CD8+ T cell responses with significant tumor reduction in metastatic melanoma lesions [24]. In addition, a nanoparticulate liposomal mRNA vaccine with sequences encoding for four class I MHC-restricted TAAs, with tetanus toxoid epitopes and targeting signal sequences intended for reaching immature dendritic cells in the lymphoid tissue, in combination with ICIs therapy in patients with resected and unresected advanced melanoma demonstrated a more effective CD4+ T cell-dominant responses, tumor killing, and a longer tumor-specific memory T-cell formation [31].

Finally, in resected pancreatic adenocarcinoma, an mRNA-based neoepitope vaccine combined with atezolizumab and chemotherapy improved recurrence-free survival and achieved a 50% overall response rate in a phase I study [53].

Another kind of nucleic acid-based vaccines is represented by DNA vaccines. These are made by closed circular DNA plasmids of bacterial origin encoding proteic antigens, and they are able to promote humoral and cellular responses restricted to target antigens, as well as to stimulate TLRs or membrane-bound receptors critical in DC priming and in B cell and natural killer cell activation, thus fostering local inflammatory reactions [14]. For example, a DNA-based vaccine based on ErbB2, an epidermal growth factor often highly expressed in some colorectal, pancreatic, endometrial, gastric, and breast cancers, has been tested in mouse models, eliciting a more powerful anti-cancer immune response compared with controls [54]. However, DNA vaccines’ delivery is more complex than that of RNA vaccines due to the higher dimensions and the nuclear localization, and thus further studies on strategies for ameliorating transfection are still needed [14].

## 4. Chimeric Proteins Stimulating the Immune System Against Tumors

An alternative approach in cancer treatment is represented by the injection of soluble molecules capable of stimulating specific immune pathways. For instance, the administration of a chimeric protein (BLS-MICA)—composed of the MICA domain fused with a molecule derived from *Brucella* spp. (BLS)—has shown promising results. This fusion protein induces the production of high titers of anti-MICA antibodies, which, in turn, neutralize MICA ligands expressed on the surface of tumor cells. This neutralization triggers Antibody-Dependent Cellular Cytotoxicity (ADCC), leading to effective tumor cell destruction [55].

## 5. Vaccines as Adjuvant to Other Immunotherapy Treatments

To overcome the challenge of delivering exogenous tumor antigens into the tumor milieu, researchers have adopted strategies to generate a large amount of endogenous cancer antigens within the tumors, thus creating “in situ cancer vaccines”. This can be achieved by inducing immunogenic cell death, which involves the apoptosis of tumor cells through chemotherapy (e.g., anthracyclines) and radiotherapy, and by upregulating the presentation of the antigens released by APCs in a proinflammatory milieu driven by a cytokine storm in the TME, which in turn facilitates the recruitment of APCs and T effector cells [18].

Nevertheless, immunogenic cell death is often insufficient to overcome the immunosuppressive TME, and adjuvant agents are needed to improve antigen presentation, repolarize immunosuppressive M2 macrophages and MDSCs, increase the CD8+/Treg ratio, and stimulate the differentiation of effector memory T cells [18].

Moreover, vaccines may upregulate the expression of PD-L1 in the tumor microenvironment, thereby turning “cold” tumors into “hot” tumors, and boosting the anticancer effects of PD-1/PD-L1 inhibitors [52].

**Figure 2:**
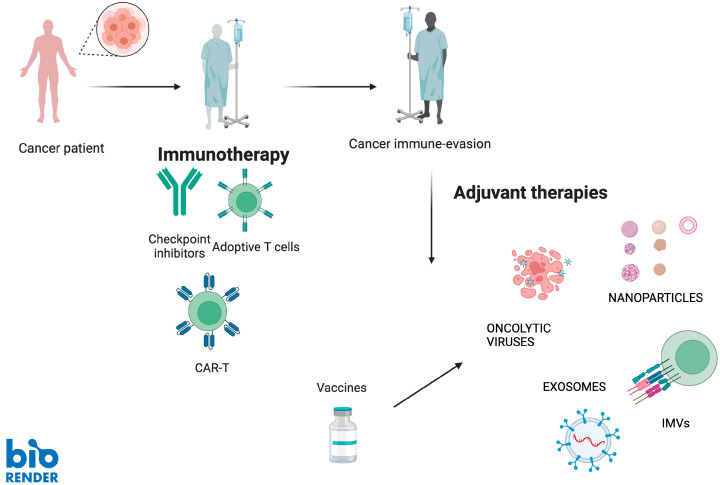
Cancer vaccines can be used in combination with other immunotherapy treatments (e.g., checkpoint inhibitors, adoptive T cells and CAR-T) to overcome cancer immune evasion mechanisms, which in turn could be used to determine tumor progression despite ongoing immune treatment. As explained in the text (in the sixth paragraph), different type of cancer vaccines have been examined in clinical trials to ameliorate patients’ objective response rates in combination with other immunotherapy medications in a sort of synergistic anti-cancer therapy. Abbreviations: chimeric antigen receptor T-cell (CAR-T), immune-modulatory vaccines (IMVs).

In this regard, in situ cancer vaccines, composed of various biomaterials such as oncolytic viruses, bacterial products, and nanomaterials, have been engineered to induce both cancer cell death and intratumoral inflammation, thereby enhancing the efficacy of concomitant anticancer treatments (Figure 2).

### 5.1. Oncolytic Virotherapy

Oncolytic virotherapy (OV) is a new type of immunotherapy using a viral vector selectively infecting and lysing tumor cells, which results in releasing tumor antigens to promote an anti-tumor adaptative immune response, while preserving normal tissues. Therefore, the combination of oncolytic viruses with other intratumoral therapies to directly alter the tumor microenvironment, thus fostering in situ vaccination effects, may be a promising future anti-cancer treatment [32].

The anti-cancer effects here are due to direct oncolysis, the induction of innate and adaptive immune responses, the alteration of the immunosuppressive TME, and the disruption of tumor blood vessels [56].

Therefore, it can be used to increase T cell migration, survival, and expansion at tumor sites, enhance APC function, reverse T cell exhaustion, and convert “cold” tumors into “hot” tumors, thereby improving the efficacy of other immunotherapy treatments [57].

Nevertheless, although oncolytic viruses have demonstrated high effectiveness against cancer, their lack of tumor specificity results in substantial off-target replication and toxicity; moreover, they may bind to various cell types, potentially preventing them from reaching the target tumor in sufficient numbers. Consequently, it is necessary either to select viruses with natural oncotropism or to genetically engineer viruses for enhanced tumor specificity, as well as to use cell carriers with tumor-homing capabilities [58].

In addition, OV exerts immunomodulatory anti-cancer activity by attracting cytotoxic T lymphocytes to the tumor and upregulating PD-L1 expression on both cancer and immune cells [59]. It also directly induces tumor cell apoptosis, thereby releasing tumor-associated antigens that are subsequently processed by APCs to stimulate a cytotoxic T lymphocyte (CTL) anti-tumor response [60].

Oncolytic viruses have been approved for various tumors such as melanoma, glioblastoma, prostate cancer, and head and neck cancer, and they could play a promising role in enhancing the effects of immunotherapy, but detailing the overall knowledge in this field lies outside the scope of this review.

### 5.2. Nanomaterial-Based Vaccines

Nanomaterial-based vaccines are increasingly used as adjuvants to enhance the efficacy of other anti-cancer immunotherapies. Among these, liposomes are the most widely employed carriers. They can be pre-loaded with chemotherapeutic agents (such as doxorubicin) and adjuvants to generate tumor antigens in situ, thereby amplifying immune responses within the TME. When combined with conventional immunotherapies, such as anti-PD-1 or anti-CTLA-4 treatments, liposomal vaccines have shown promise in preclinical models of colon cancer and melanoma [33]. Liposomal formulations can also be engineered to include molecules that counteract the immunosuppressive TME and improve local antigen presentation, such as Toll-like receptor agonists, cyclic GMP-AMP (cGAMP), and bacterial membrane components [61].

Other notable nanomaterial-based carriers include inorganic nanoparticles such as silica and alum. These materials stimulate local inflammatory responses by delivering cytokines such as IL-12, IL-2, IL-15, and IFN-γ, thereby promoting dendritic cell (DC) recruitment and T cell priming [62,63]. They can also be pre-loaded with chemotherapeutics or radiation particles to induce immunogenic cell death, further enhancing the immune-stimulatory effects of other therapies [64].

Another novel anti-cancer vaccines approach uses soluble antigens mixture released into cell culture supernatants of several melanoma cell lines, to induce both antibody and T cell responses to the antigens in the vaccines. Even though a small randomized placebo-controlled phase II trial showed significant improvement in recurrent free survival, no phase III trials are currently available that support this hypothesis [65].

## 6. Other Kinds of Vaccines in Immunotherapy Adjuvant Setting

Exosomes are extracellular vesicles capable of transferring various intracellular molecules—including antigens and MHC complexes—to adjacent or distant cells. Exosomes play a dual role: they facilitate communication between immune cells and present tumor-specific antigens to the immune system, thereby counteracting the immune evasion mechanisms employed by cancer cells [4].

Exosomes derived from immune cells within the TME have distinct functions. NK cell-derived exosomes contain cytotoxic proteins (e.g., perforin, Fas ligand, granzyme A/B, and granulysin) that directly kill cancer cells. They also transport cytokines like IL-15 and tumor-suppressive microRNAs that inhibit tumor growth. M1 macrophage-derived exosomes enhance local inflammation and promote the conversion of M0 macrophages into pro-inflammatory, anti-tumor phenotypes by releasing cytokines such as IL-6, IL-12, and TNF-α [4]. Conversely, tumor-derived exosomes can facilitate immune escape by increasing the expressions of PD-L1 and CD73 and by secreting immunosuppressive molecules (e.g., lactic acid), which downregulate costimulatory molecules (CD28, CD80) on T cells and promote immunotherapy resistance [66,67]. Despite these complexities, tumor-derived exosomes hold promise as vaccines. By loading MHC-I and tumor antigens onto their surface, tumor-derived exosomes can effectively induce T cell-mediated anti-tumor responses. Preclinical studies in melanoma and colon cancer models have shown that TDE-based vaccines prolong survival, delay tumor onset, provide long-term immune memory, and reduce metastasis [68].

A specialized subgroup of exosomes, known as dexosomes, are secreted by DCs. Dexosomes contribute to antigen presentation by incorporating MHC–antigen complexes, which are subsequently transferred to naïve DCs, triggering both innate and adaptive immune responses [3]. Once administered, dexosome vaccines efficiently reach lymph nodes, where they are internalized by resident DCs. This uptake stimulates T cells through the expression of costimulatory molecules (CD54, CD80, CD86) and integrins (CD11b/CD18, CD11a/CD18), while also delivering DC-modulating microRNAs (miRNAs) [3]. In addition to activating T cells, dexosomes can stimulate B cells to produce antigen-specific antibodies, thereby coordinating both humoral and cellular immune responses [69]. Second-generation dexosome therapies have further enhanced anti-tumor effects by promoting NK cell function via NKp30 receptor engagement and BAG6 ligand expression, which together trigger TNF-α and IFN-γ secretion [70].

Emerging platforms such as antigen self-presentation and immunosuppression reverse (ASPIRE) leverage DC-derived membranes loaded with multiple costimulatory signals (CD80/CD86) and anti-PD-1 antibodies to strengthen T cell-mediated immunity [71]. Combination therapies involving dexosomes and immune checkpoint blockades (e.g., PD-1/PD-L1 or CTLA-4 inhibitors) are under investigation for their potential to overcome TME immunosuppression and enhance T cell activation. However, the optimization of molecular and subcellular components remains ongoing, with only two clinical studies completed to date showing promising preliminary results [70,72].

### Immune-Modulatory Vaccines (IMVs)

Immune-modulatory vaccines (IMVs) work by activating antiregulatory T cells (anti-Tregs), a subgroup of T cells that specifically target regulatory immune cells, thereby mitigating the immunosuppressive TME. IMVs not only facilitate the direct cytotoxic targeting of tumor cells, but also remodel the TME into a proinflammatory environment that restricts tumor progression. This remodeling includes reprogramming TAMs from an immunosuppressive M2 phenotype to an M1-like phenotype, which supports anti-tumor T cell responses and enhances antigen presentation. Additionally, IMVs can convert cancer-associated fibroblasts (CAFs) into immunocompetent fibroblasts, further promoting anti-tumor immunity.

One example is a TGF-β-based IMV developed to control tumor growth in murine models of pancreatic cancer. This vaccine targets both immunosuppression and fibrosis within the TME. Vaccine-induced T cells not only directly kill tumor cells, but also indirectly modulate the immunosuppressive environment by targeting M2 macrophages and CAFs [27,73]. Another promising approach involves indoleamine 2,3-dioxygenase (IDO)-based vaccines. A phase I clinical trial demonstrated that IDO-targeting monotherapy resulted in a median OS of 26 months and prolonged disease progression-free survival of up to six years in lung cancer patients when used as maintenance therapy [74].

The combination of IMVs with ICIs is emerging as a particularly appealing strategy. IMVs can induce novel T cell activation, foster local inflammation, and modulate the TME to create an immune-favorable environment that enhances the anti-tumor efficacy of ICIs. This combination approach was evaluated in a phase II clinical trial involving patients with metastatic melanoma, wherein the therapy achieved an 80% objective response rate [34,75]. An ongoing multicenter phase III trial (NCT05155254) is investigating the efficacy of an IDO/PD-L1 vaccine in combination with pembrolizumab compared to pembrolizumab alone. Preliminary results indicate promising improvements in progression-free survival and safety profiles, consistent with outcomes observed in preclinical models.

Beyond ICIs, combining IMVs with other immunotherapies—such as ACT or traditional cancer vaccines—represents a promising avenue for enhancing anti-tumor immunity. While in vitro models have shown encouraging results, further in vivo studies are necessary to fully assess the clinical applicability of these approaches [27] (Figure 2).

## 7. Limitations of Cancer Vaccines as Immunotherapy

Cancer vaccines face several limitations that affect their clinical applicability. These challenges include peripheral and central immune tolerance to tumor antigens, the risk of inducing autoimmunity against normal tissues, and the rapid mutational evolution of tumors, which allows them to evade T cell-mediated immune responses.

Regarding immune tolerance, neoantigens—which are tumor-specific antigens—are less likely to induce immune tolerance or trigger autoimmune reactions due to their absence on normal cells. However, the development of neoantigen-based vaccines remains challenging because of the limited and often variable expression of these antigens on the surface of cancer cells [27].

In addition, off-target effects arising from antigens shared on both tumor and normal cells, dominant antigens that promote tumor immune escape or limit T cell responses, and the suboptimal selection of vaccine adjuvants for antigen processing and presentation, represent other important limitations to the clinical effectiveness of cancer vaccines [76].

Furthermore, various vaccine platforms—such as peptide-based, cell-based, RNA, DNA, and viral vector-based vaccines—present inherent challenges that limit their clinical utility, such as rapid degradation by host enzymes within the TME, which can reduce their efficacy [77]; low intrinsic immunogenicity leading to naturally poor immunogenicity, necessitating high doses of genomic material or the use of potent adjuvants to elicit sufficient immune responses [27]; and other mechanisms of resistance [78]. Key resistance mechanisms include the following:

- The downregulation of HLA molecules, reducing antigen presentation to T cells;
- Decreased neoantigen load, limiting the number of recognizable tumor antigens;
- Aberrations in key signaling pathways, affecting immune activation;
- Adaptive immune system modifications that impair long-term immune responses;
- Innate immune evasion mechanisms, leading to immune cell dysfunction and destruction.

Given all these limitations, the use of vaccines as a cancer prevention strategy for solid tumors in high-risk individuals, such as genetically predisposed subjects or pre-malignant lesion carriers, in clinical settings may be important to optimize vaccine efficacy [7].

## 8. Economic Evaluation of Cancer Immunotherapy

The rising prominence of immunotherapies for solid tumors, including monoclonal antibodies (mAbs) and therapeutic cancer vaccines, has prompted critical evaluations of their economic impact; so far, most of the data regarding solid tumors have been reported for monoclonal antibodies. The systematic review by Geynisman et al. is the only one to have analyzed the cost-effectiveness of a cancer vaccine (e.g., Sipuleucel-T) that was available in 2014 for the treatment of castration-resistant prostate cancer (now unavailable in clinical practice due to the aforementioned reasons), and remarked on an incremental cost–effectiveness ratio (ICER) of nearly USD 290,000 per quality-adjusted life (QALY), raising concerns about its affordability despite its modest survival benefits. Conversely, rituximab (a monoclonal antibody) was associated with favorable ICERs in multiple studies, reinforcing its role as a benchmark for cost-effective immunotherapy, particularly in the treatment of non-Hodgkin lymphoma. Other agents showed limited cost-effectiveness due to high ICERs that exceeded widely accepted thresholds (e.g., USD 50,000–100,000 per QALY).

A central theme emerging from the literature is the lack of predictive biomarkers to guide patient selection, which limits the clinical and economic efficiency of these therapies. Inappropriate patient selection not only reduces efficacy, but also exacerbates healthcare costs. Moreover, the pricing strategies of pharmaceutical companies—often based on the highest-priced competitor—contribute to unsustainable cost escalations.

Despite the financial burden, the clinical value of immunotherapies remains significant, particularly for patients who achieve durable responses.

In conclusion, while cancer immunotherapies represent a paradigm shift in oncologic care, their cost-effectiveness remains variable and context-dependent. Moreover, further analyses are needed in the field of cancer vaccines, which are still under-investigated from the point of view of their economic impact on the healthcare system. Future research should prioritize the development of predictive tools, rational pricing models, and real-world data to refine the economic framework guiding immunotherapy deployment [79].

## 9. Conclusions

Several studies have shown that a low pretreatment lymphocyte count is associated with poor survival outcomes in patients with solid tumors undergoing immune-based therapies [80,81]. In contrast, patients with preexisting immunity to tumor neoantigens exhibit improved clinical responses to immunotherapy [82]. Based on these findings, combining ICIs with agents that induce a sustained neoantigen-specific T cell response represents a promising strategy for treating advanced solid tumors that are refractory to conventional therapies.

Cancer vaccines, in this context, may emerge as key adjuvant immunotherapies, as they can recruit and activate T cells specific to tumor-associated antigens. This capability allows vaccines to overcome the limitations of ICIs, especially in targeting the “cold” tumors—those with poor immunogenicity—commonly observed in advanced and metastatic solid cancers. As previously discussed, these vaccines enhance the host’s immune defense by promoting local tumor-neoantigen presentation, which helps restore the population of effector T cells and bolsters the efficacy of other immunotherapies in preclinical models.

Importantly, the neoantigen-specific T cells induced by vaccines are more likely to persist, circumvent physiological immune tolerance, and minimize off-target effects, thereby improving overall safety [83]. Additionally, cancer vaccines may enhance the antitumor activity of ICIs by reversing the immunosuppressive TME, as demonstrated with certain immunomodulatory vaccines [27].

However, several challenges remain in optimizing vaccine efficacy, as the selection of the most immunogenic and tumor-specific tumor antigens remains a key hurdle, as do the choices of biomaterials and delivery methods: there are several ongoing trials exploring this issue (Table 2). The ideal delivery platform should maximize immune activation while ensuring safety and tolerability. In addition, reliable radiological and biochemical markers for evaluating objective response rates and the proportion of activated effector T cells in combination therapies (ICIs plus vaccines) are still underdeveloped [16]. Importantly, host factors, including sex, age, and the gut microbiome, can contribute to modulate cancer vaccines effectiveness. None of these have been adequately characterized, and no biomarkers for vaccine efficacy have been formally validated. Therefore, future studies regarding patients’ features are needed to elaborate scores to predict the effectiveness or failure of cancer vaccines in advanced tumor settings [7].

Addressing these challenges is essential for translating these innovative therapies into effective clinical solutions for patients with hard-to-treat cancers.

## Figures and Tables

**Table 1:** Cancer vaccines’ composition and application in clinical studies.

Composition	Effect	Clinical Example	Target Tumor	Clinical Application
Exosomes [3,4]	- enhancing APC processing - reversing immunosuppressive TME	T-Vec	Melanoma	Adjuvant to ICIs
Cell-based [7]	- priming effector T cells	DCs	Prostate cancer	Primary immunotherapy alone
Protein-based [29]	- enhancing natural killer activity	Irradiated tumor cells	Melanoma	Primary immunotherapy alone
Nucleic acid-based [30]	- enhancing APC processing	Neoantigen v	Cervical cancer	Primary immunotherapy alone
Soluble molecules [31]	- induction of anticancer antibodies	mRNA	High tumor mutational burden cancers	(ongoing trials)
Oncolytic viruses [32]	- direct cancer cells oncolysis - induction of innate and adaptive immune responses - reversing immunosuppressive TME	KRAS	melanoma	Adjuvant to ACT
Nanomaterial-based [33]	- increasing local tumor antigen release - stimulate local inflammation	hTERT	Pancreatic cancer	Adjuvant to ICIs
Immune-modulatory vaccines [34]	- reversing immunosuppressive TME	PROSTVAC	Prostate cancer	Adjuvant to ICIs

Abbreviations: dendritic cell (DC), messenger RNA (mRNA), antigen-presenting cell (APC), Kirsten Rat Sarcoma viral oncogene homolog (KRAS), adoptive cell therapy (ACT), human telomerase reverse transcriptase (hTERT), checkpoint inhibitor (ICI), talimogene laherparepvec (T-Vec).

**Table 2:** Future perspectives in clinical research on cancer vaccines.

Clinical Trial ID	Target (Peptide)	Indication	Phase	Study Completion Date
NCT03559413	Patient-individualized peptide	Acute lymphoblastic leukemia	1, 2	2023
NCT04688385	Multipeptide vaccine	Leukemia	1	2024
NCT05475106	Neoantigen peptide	Different type of cancer	1	2024
NCT04842513	Multipeptide	Glioblastoma	1	2025
NCT04270149	ESR1 peptide Vaccine	Breast cancer	1	2026
NCT06949410	Peptide Vaccine	Breast cancer	1	2030
NCT06932861	Personalized Vaccine	Rhabdomyosarcoma	Early 1	2028
NCT06789198	Peptide Vaccine	Fibrolamellar hepatocellular carcinoma	1	2028

## Abbreviations

The following abbreviations are used in this manuscript: checkpoint inhibitors (ICIs), adoptive cell therapy (ACT), tumor microenvironment (TME), regulatory T cells (Tregs), myeloid-derived suppressor cells (MDSCs), antigen-presenting cells (APCs), major histocompatibility complex (MHC), dendritic cells (DCs), cytotoxic T lymphocytes (CTLs), tumor-associated M2 macrophages (TAMs), anti-regulatory T cells (anti-Tregs), natural killer (NK), prostate acid phosphatase (PAP), Food and Drug Administration (FDA), tumor-infiltrating lymphocytes (TILs), tumor-specific antigens (TSAs), tumor mutation burden (TMB), interferon (IFN), oncolytic viruses (OVs), Talimogene laherparepvec (T-Vec), herpes simplex virus type 1 (HSV-1), prostate-specific antigen (PSA), oncolytic myxoma virus (vMyx), measles virus (MeV), hepatocellular carcinoma (HCC), cyclic GMP-AMP (cGAMP), microRNAs (miRNAs), antigen self-presentation and immunosuppression reverse (ASPIRE), immune-modulatory vaccines (IMVs), cancer-associated fibroblasts (CAFs), indoleamine 2,3-dioxygenase (IDO), granulocyte–macrophage colony-stimulating factor (GM-CSF), human telomerase reverse transcriptase (hTERT), T cell receptor (TCR), tumor-associated antigen (TAA), messenger RNA (mRNA).
